# Objective Perfusion Assessment With Near Infrared Fluorescence for Guidance of Bowel Resection Margins Following Superior Mesenteric Artery Thrombosis: A Case Report

**DOI:** 10.1016/j.ejvsvf.2025.08.006

**Published:** 2025-09-05

**Authors:** Roderick C. Peul, Floris P. Tange, Joost R. van der Vorst

**Affiliations:** Department of Surgery, Leiden University Medical Centre, Leiden, the Netherlands

**Keywords:** Embolectomy, Indocyanine green, Near infrared fluorescence imaging, Perfusion assessment, Quantification, Superior mesenteric artery

## Abstract

**Objective:**

Acute occlusion of the superior mesenteric artery (SMA) results in extensive bowel ischaemia, with mortality rates approaching 70%. Management involves acute revascularisation, typically via embolectomy, and intra-operative evaluation to distinguish viable from non-viable intestinal tissue. Near infrared fluorescence (NIRF) imaging with indocyanine green (ICG) enables real time, minimally invasive assessment of tissue perfusion. This case report illustrates the usability of NIRF imaging to guide surgical decision making for bowel preservation and patient survival in a clinical situation where research is limited.

**Report:**

A 40 year old male presented, in June 2023, with severe postprandial abdominal pain. Imaging revealed a non-occlusive thrombus in the SMA, which together with clinical deterioration resulted in an emergency embolectomy. Intra-operative NIRF imaging with ICG identified malperfusion of a jejunal segment. A second NIRF assessment post-embolectomy confirmed persistent malperfusion, guiding resection with a primary anastomosis. Quantitative fluorescence analysis confirmed the intra-operative findings of malperfusion and demonstrated potential utility for objective perfusion assessment to resect non-viable tissue and limit viable tissue resection. The patient recovered uneventfully and was discharged on post-operative day five.

**Discussion:**

This case highlights the use of NIRF imaging as a safe, low cost technique for evaluating bowel perfusion during SMA embolectomy. By selecting adjacent regions of interest, surgeons can compare objective perfusion curves intra-operatively and select optimal anastomosis sites. Quantified parameters further aid decision making, showing clear percentage differences in perfusion between viable and ischaemic tissue. Although standardised cut off values are lacking, combining clinical evaluation, subjective assessment, and quantitative data offers a patient tailored approach to optimising resection margins.

## HIGHLIGHTS

• Current assessment tools lack accuracy in predicting ischaemia during superior mesenteric artery (SMA) occlusion.
• Fluorescence imaging aids in bowel perfusion assessment during SMA embolectomy.
• Quantified perfusion curves and parameters enhance resection margin determination.
• Fluorescence imaging helps preserve viable tissue and maintain bowel function.

## INTRODUCTION

The superior mesenteric artery (SMA) is responsible for perfusion from the distal part of the duodenum to approximately two thirds of the transverse colon.^1^ Acute occlusion of this artery results in ischaemia of a substantial part of the bowel, with mortality rates up to 50% when diagnosed within 24 hours. This rate increases to 70% when this period is exceeded.^2^ Intervention focuses on reversing the infarction by embolectomy. Intra-operative assessment of the intestinal tissue is critical to determine whether ischaemic damage is reversible or if resection of non-viable segments of the intestine is necessary.^3^ When resection is inevitable, surgeons must define the optimal extent of tissue removal to ensure patient survival, while preserving as much intestine as possible to maintain adequate gastrointestinal function. Near infrared fluorescence (NIRF) imaging with indocyanine green (ICG) has shown feasibility in assessment of tissue perfusion across various organ systems, while imposing minimal additional burden on the patient.^4^, ^5^, ^6^ This report describes a case in which NIRF imaging was effectively employed to assess intestinal perfusion after SMA thrombosis, contributing to both patient survival and the preservation of intestinal function. Informed consent is available on request.

## REPORT

A 40 year old male patient presented to the emergency department of a community hospital in the Netherlands in June 2023 with severe, diffuse postprandial abdominal pain radiating to the back. The medical history included Raynaud syndrome, hypertension, and a >30 pack year smoking history. Medication consisted of omeprazole. No allergies were reported. Computed tomography angiography (CTA) of the abdomen, pelvis, and lower extremities showed a non-occlusive thrombus in the SMA, a floating thrombus in the common iliac artery, and occlusion of the left distal tibioperoneal trunk. Echocardiography did not indicate a cardiac source for the embolus. CT abdomen did not indicate signs of bowel wall thickening, free air, fluid collections, or oedema. Laboratory results showed elevated C reactive protein and lactate. The patient was started on analgesics and 20 000 units/24 hours of heparin, after which he was transferred to an academic hospital, where he remained stable. On the second night following transfer, the patient's condition deteriorated with the recurrence of severe abdominal pain. Urgent SMA embolectomy was scheduled.

At laparotomy, an oedematous segment of the jejunum was identified approximately 10 cm distal to the ligament of Treitz (Appendix 1, black arrows). Additional perfusion assessment of the jejunum was conducted by intravenous administration of ICG (5 mg bolus) with immediate visualisation using the Quest Spectrum fluorescence imaging system (Quest Medical Imaging, Wieringerwerf, the Netherlands). The subjective fluorescence pattern demonstrated reduced ICG inflow in the compromised jejunum compared with the rest of the tissue (Fig. 1A).

**Figure 1:**
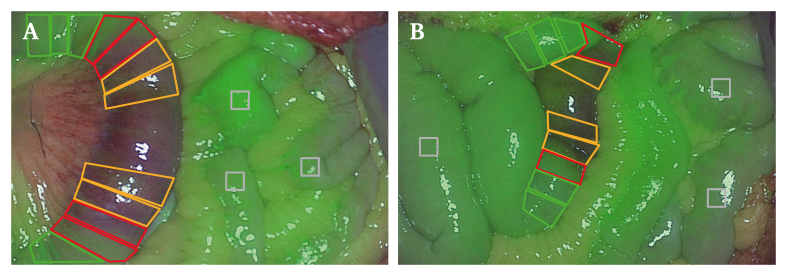
Near-infrared fluorescence measurement of the jejunum A before and B after embolectomy. The coloured boxes are subjectively selected regions of interest from which time intensity curves are extracted. Green = adequate perfusion, orange = dubious perfusion, red = ischaemic region, grey = control area.

Following evaluation of the jejunum at risk, a subjective assessment of the remaining intestine was performed, revealing no further perfusion defects. SMA arteriotomy showed an organised thrombus. A second NIRF perfusion assessment with ICG was performed 15 minutes after successful embolectomy (Fig. 1B), revealing no improvement in perfusion within the compromised tissue.

Given the minimal chance of recovery of the affected jejunal segment, resection was deemed necessary. To safeguard the patient's outcomes in terms of both survival and intestinal function, the amount of resected tissue was guided by a combination of clinical judgement and the subjective fluorescence assessment. Subsequently, an end to end anastomosis was created and the abdominal wall was closed in layers.

Post-operatively, the fluorescence measurements were quantified to provide objective perfusion parameters of the affected tissue. Time intensity curves derived from subjectively selected, adjacent regions of interest provided objective evidence of impaired perfusion before embolectomy compared with control tissue (Appendix 2). The perfusion curves and parameters after embolectomy indicated persistent malperfusion of the affected segment (Fig. 2, Table 1).

**Figure 2:**
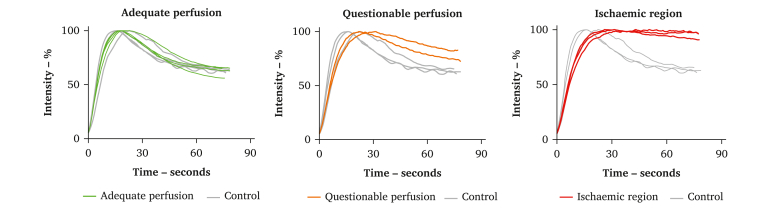
Normalised time intensity curves for regions of interest deemed to have adequate perfusion, questionable perfusion, or ischaemia after embolectomy. The grey curves in each graph represent control regions of interest corresponding to tissue expected to be unaffected.

**Table 1:** Perfusion parameters per region of interest compared with the mean values of the control tissue.

	Tmax	Δ to control	MaxSlope	Δ to control	AUC30	Δ to control
Control ROI (mean of *n* = 3)	**17.42**	**-**	**10.93**	**-**	**83.92**	**-**
Adequate perfusion 1	17.25	**−1%**	11.08	**+1%**	84.06	**<+1%**
Adequate perfusion 2	17.25	**−1%**	11.58	**+6%**	84.27	**<+1%**
Adequate perfusion 3	17.50	**<+1%**	11.98	**+10%**	85.00	**+2%**
Adequate perfusion 4	17.00	**−2%**	9.71	**−11%**	84.95	**+1%**
Adequate perfusion 5	21.00	**+21%**	10.53	**−4%**	89.38	**+7%**
Questionable perfusion 1	21.50	**+23%**	9.20	**−16%**	91.55	**+9%**
Questionable perfusion 2	30.50	**+75%**	8.37	**−23%**	93.20	**+11%**
Ischaemic region 1	31.00	**+78%**	8.65	**−21%**	97.77	**+17%**
Ischaemic region 2	42.00	**+141%**	7.73	**−29%**	98.88	**+18%**
Ischaemic region 3	30.00	**+72%**	8.74	**−20%**	96.66	**+15%**

ROI = region of interest, Tmax = time until maximum fluorescence intensity is reached (sec), MaxSlope = maximum inflow rate (%/sec), AUC30 = remaining fluorescence intensity 30 seconds after Tmax (%).

On the first post-operative day, the patient reported wound related pain, while the diffuse abdominal pain had resolved. Abdominal CT revealed no abnormalities except for oedema at the jejunal resection site. The patient was discharged on post-operative day five and achieved full recovery.

## DISCUSSION

This case highlights the utility of NIRF imaging with ICG for resection margin assessment in a patient with suspected bowel ischaemia during SMA embolectomy. Accurate assessment of tissue viability is difficult in these patients because conventional evaluation methods lack sufficient predictive accuracy. This often results in broad resection margins that aim to safeguard patient survival but sacrifice viable bowel tissue. NIRF imaging has emerged as a safe and user friendly intra-operative technique for evaluating the entire bowel to identify compromised segments.^7^ However, its clinical utility remains limited due to the subjective interpretation of the fluorescence signal, rendering the determination of precise resection margins largely dependent on the judgement of the surgical team.

To address this limitation, quantification can be integrated into the technique. Vaassen *et al.* demonstrated the potential of quantified perfusion parameters as time until maximum intensity and maximum inflow rate in differentiating between larger segments of viable and non-viable tissue during mesenteric artery occlusion.^8^ In this case report, these findings were directly applied to the area of resection. Time intensity curves clearly illustrated distinct perfusion patterns between regions at risk and the control tissue. A delayed time to peak intensity and an absence of outflow were especially observed for the ischaemic region compared with the control tissue, which was further substantiated in a clear percentual difference, as shown in Table 1. This quantitative approach supports surgeons by providing objective metrics to assess perfusion in areas considered for anastomosis.

This case had certain limitations. As the affected bowel was resected, it cannot be excluded that the tissue might have recovered without resection. Furthermore, the measurement is time dependent. Following embolectomy, the tissue undergoes reperfusion, which is expected to exhibit a distinct fluorescence perfusion pattern immediately after revascularisation compared with that observed after a short period of physiological adaptation to the restored blood flow.^9^ It would therefore seem logical to allow a short interval of reperfusion before performing the fluorescence assessment, in order to obtain a more representative evaluation of tissue viability. However, the optimal duration of this reperfusion interval remains undetermined. In cases where second look surgery is performed, a third NIRF measurement could provide valuable information about the tissue recovery after an extended period of reperfusion. Finally, initial resection margins were not determined by clinical evaluation alone. Future studies should ideally incorporate the individual resection margins based on clinical assessment, subjective NIRF imaging, and quantified NIRF parameters.

Currently, there are insufficient data to adhere to cut off values of fluorescence based perfusion parameters for resection demarcation. Powered clinical trials should demonstrate the feasibility of such threshold values for assessing the extent of bowel ischaemia in patients suffering from acute mesenteric ischaemia. However, as demonstrated in this case report, a combination of clinical evaluation, subjective fluorescence assessment, and objective time intensity curves with derived perfusion parameters provides the surgeon with a more patient tailored approach for evaluating the affected intestinal tissue. This facilitates an improved balance between patient survival and post-operative intestinal functionality.

## Declaration of generative ai and ai assisted technologies in the writing process

During the preparation of this work the author(s) used ChatGPT in order to improve language use and readability. After using this tool, the author(s) reviewed and edited the content as needed and take(s) full responsibility for the content of the publication.

## Conflict of interest

None.

## Figures and Tables

**figs1:**
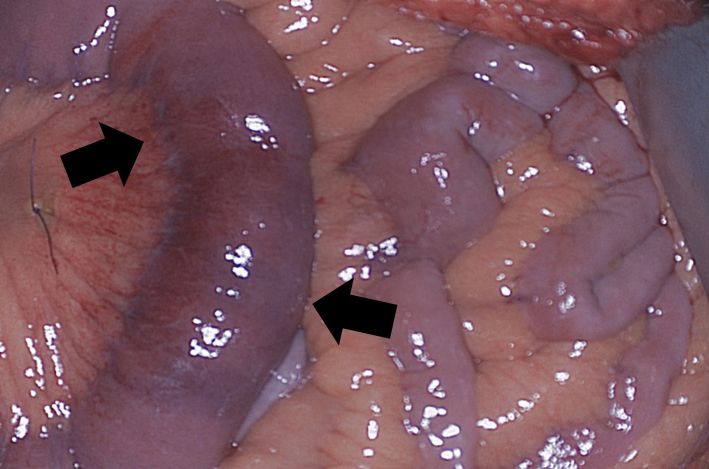
Oedematous segment of the jejunum.

**figs2:**
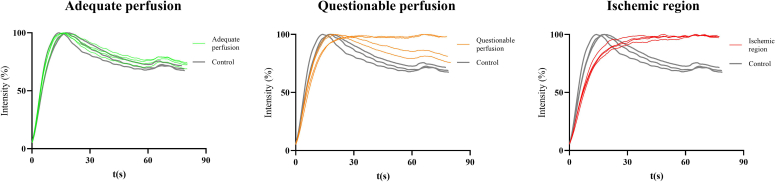
Normalised time intensity curves for regions of interest deemed to have adequate perfusion, questionable perfusion or ischaemia before embolectomy. The grey curves in each graph represent control regions of interest corresponding to tissue expected to be unaffected.
